# GlioM&M: Web-based tool for studying circulating and infiltrating monocytes and macrophages in glioma

**DOI:** 10.1038/s41598-020-66728-w

**Published:** 2020-06-18

**Authors:** Erik R. Abels, Sybren L. N. Maas, Eric Tai, David T. Ting, Marike L. D. Broekman, Xandra O. Breakefield, Joseph El Khoury

**Affiliations:** 1 Departments of Neurology and Radiology, Massachusetts General Hospital, and Harvard Medical School, 02129 Boston, Massachusetts USA; 2 Department of Neurosurgery and Department of Pathology, UMC Utrecht Brain Center, University Medical Center, Utrecht University, Utrecht, 3584 CX The Netherlands; 3 Cancer Center, Massachusetts General Hospital, and Harvard Medical School, Boston, 02114 Massachusetts USA; 4 Department of Neurosurgery, Leiden University Medical Center, Leiden, 2300 RC The Netherlands; 5 Department of Neurosurgery, Haaglanden Medical Center, The Hague, 2512 VA The Netherlands; 6 Center for Immunology & Inflammatory Diseases, Massachusetts General Hospital, and Harvard Medical School, Boston, 02129 Massachusetts USA

**Keywords:** Data publication and archiving, CNS cancer, Innate immune cells, Tumour immunology

## Abstract

Monocytes, macrophages and microglia make up a large part of the glioma environment and have an important role in maintaining and propagating glioma progression. Targeting these cells to inhibit their tumor-promoting effect and reprogramming them into an anti-tumor phenotype is a potential therapeutic approach for glioma. In this study we analyzed the transcriptomes of eight different monocyte subgroups derived from the brain and the blood of glioma-bearing mice. We compared the expression profile of blood-derived monocytes versus tumor-infiltrating monocytes and found increased expression of both pro- and anti-inflammatory pathways in tumor infiltrating monocytes. To help disseminate these datasets, we created a user-friendly web-based tool accessible at www.glioma-monocytes.com. This tool can be used for validation purposes and to elucidate gene expression profiles of tumor-interacting monocytes and macrophages as well as blood-derived circulating monocytes. This tool can also be used to identify new markers and targets for therapy in these different cell populations.

## Introduction

Glioblastomas (GBs) are the most common and lethal primary brain tumors and are characterized by their highly aggressive nature including rapid tumor growth, diffuse invasiveness and resistance to therapy^1,2^. GBs consist of a heterogeneous population of malignant cells and various types of stromal cells, which all contribute to tumor formation, progression and response to treatment^3–5^. GB cells have been shown to affect endogenous central nervous system (CNS) cells, such as microglia, astrocytes, oligodendrocytes, endothelial cells and neurons as well as infiltrating monocytes/macrophages^3^. Tumor-cell production and secretion of chemokines and cytokines—including growth and angiogenic factors and extracellular matrix modifying enzymes, as well as RNA and proteins within extracellular vesicles—create a favorable tumor microenvironment^3,6^.

In glioma, the microenvironment, including the blood-brain barrier, is severely disrupted resulting in the infiltration of myeloid-derived innate immune cells^6^. In established glioma tumors, a large proportion of the immune cells are microglia supplemented with infiltrating monocytes recruited from the blood circulation that subsequently can differentiate into macrophages^7^. In the circulating blood, two subtypes of monocytes exist that can be differentiated based on the expression level of Ly6C. Ly6C^low^ monocytes are characterized as “patrolling monocytes” and remain in the bloodstream^8^. The main function of these monocytes is to monitor the blood vessel walls and initiate vessel repair^9^. Ly6C^high^ monocytes have the capacity to sense and extravasate into tissue sites of inflammation and injury, including tumors^10–12^. Once within a glioma tumor, the Ly6C^high^ monocytes are activated and due to different factors secreted from tumors, a portion of infiltrating cells differentiate into macrophages^10^. Recently it has been shown that some infiltrating monocytes can also remain as monocytes within the tissue, where they acquire antigen-presenting functions^13^.

The influx of blood-derived monocytes and recruitment of microglia into a tumor tends to support tumor progression to more malignant grades^14^. Due to the overlap in cellular markers in human tissue, these two cell types are commonly grouped together as tumor associated myeloid cells (TAMs)^3,5,7^. TAMs are recruited to the tumor site through tumor secretion of cytokines and chemokines, including ATP, CSF-1, CCL2, GDNF, GM-CSF HGF/SF, MCF-3, SDF-1, TNF and VEGF^6,15^. To support tumor growth, TAMs secrete angiogenic factors such as CXCL2, EGF and VEGF, to induce neovascularization which is required to keep the tumor supplied with nutrients during its expansive growth^15,16^. GBs are characterized by a high level of tumor cell invasiveness, which is supported by extensive tissue remodeling. TAMs contribute to this process by the secretion of matrix metalloproteases (MMPs). For example, MMP2 degrades the brain extracellular matrix facilitating tumor cell migration^17^. In addition, TAMs have been shown to produce low levels of pro-inflammatory factors, but do not express T-cell co-stimulatory molecules, such as CD80 and CD86, indicating an inability to induce an immune response^18^. To better understand the underlining mechanisms of differentiation, immunosuppression, angiogenesis and tumor support by TAMs we profiled the RNA expression of different infiltrating monocytes and macrophages and compared them to circulating monocytes.

Understanding these types of cells is important since the focus of glioma therapy is shifted towards targeting the microenvironment as well as the tumor cells. Since the TAMs play a crucial role in maintaining the tumor, in the form of immune suppression and angiogenesis, inhibiting these features can yield a successful (adjuvant) therapy. Finding a suitable target in these cells can be achieved by studying the transcriptome. With the costs of RNA sequencing going down over the recent years, whole transcriptome analysis has become more accessible. While data submission into GEO database is becoming mandatory, retrieving data and accessing it is not user-friendly. Online resources, such as http://www.brainrnaseq.org/ or https://www.proteinatlas.org/, are examples of user-friendly, freely available datasets^19–21^. These can be used to analyze expression of specific genes in different tissues or CNS cell types. While these databases supply a baseline for gene expression levels in normal physiological setting, we have supplemented this by studying the RNA expression profile of the different monocyte subpopulations in a pathological setting.

We analyzed the RNA expression profile of different cell populations, including circulating Ly6C^high^ and Ly6C^low^ blood-derived monocytes, glioma monocytes and glioma macrophages divided into CCR2^high^ and CCR2^low^ subtypes. For the populations localized in the brain we further divided these populations into subpopulations based on the uptake of tumor-derived cell-membrane particles. This results in eight different populations of which drastic changes in RNA expression are observed once the cells had entered the glioma environment. Further analysis of specific cytokine pathways indicated a high level of activation of different cytokine-associated gene sets in the glioma-infiltrating cells. All these transcriptomic data have been compiled in a user-friendly web-based tool accessible at www.glioma-monocytes.com, making this data publicly available. This tool can be used for validation purposes and to elucidate gene expression profiles of tumor interacting-monocytes and macrophages as well as blood-circulating monocytes.

## Results

### Isolation of blood and brain infiltrated monocytes and macrophages in a glioma mouse model

**Figure 1:**
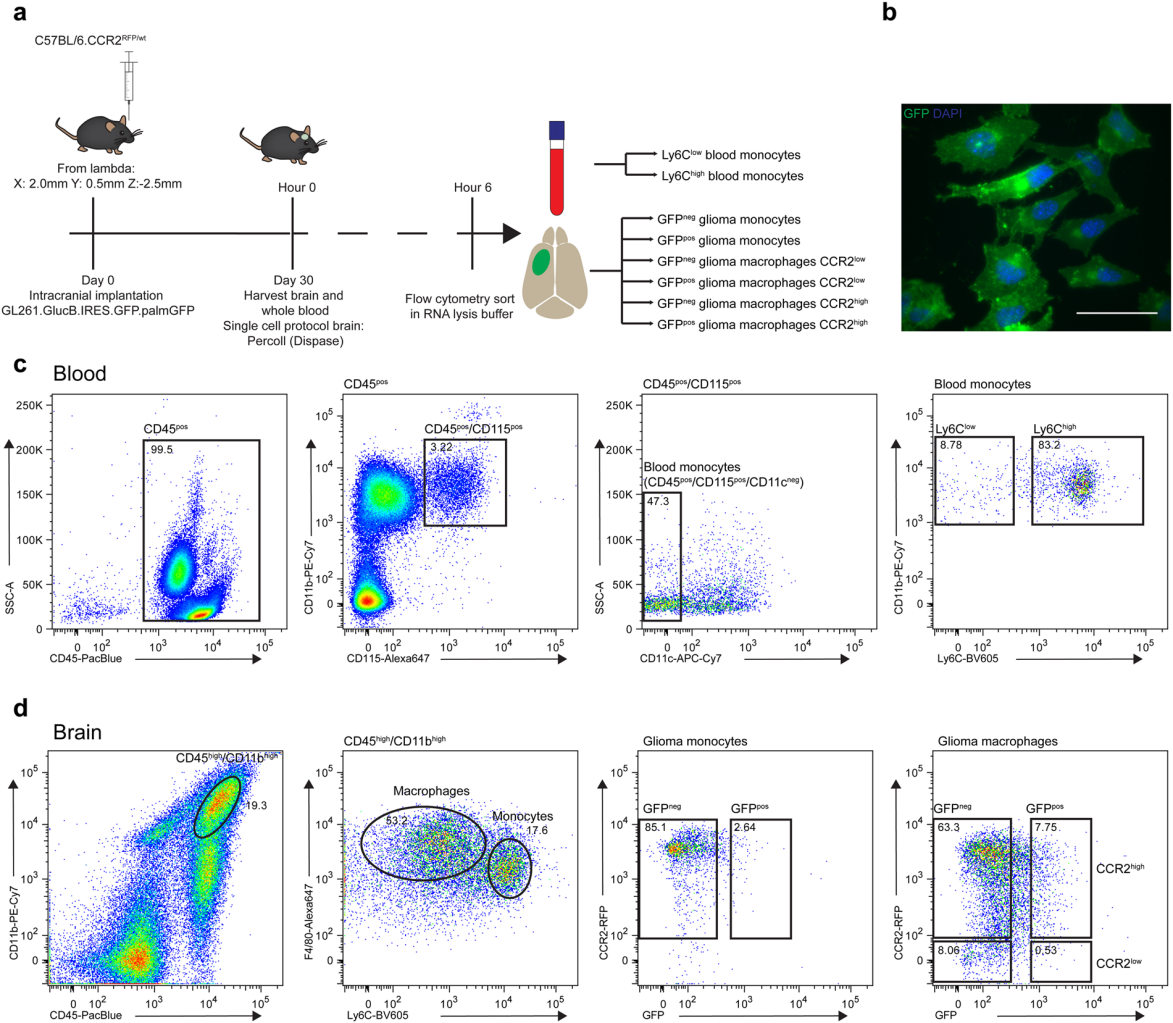
Isolation of blood and brain infiltrated monocytes and macrophages from glioma mouse model. (**a**) Schematic overview of experiment shows timeline and methods used. (**b**) Glioma cell line GL261.BpalmGFP was injected intracranially into syngeneic mouse to establish brain tumor model. Scale bar 50 µm (**c**) Glioma monocytes and macrophages were separated from microglia by FACS based on the expression level of CD45 and CD11b. Monocytes were isolated based on the presence of F4/80 and high expression of Ly6C, macrophages were further divided into CCR2 high and low. (**d**) Blood from tumor-bearing mice was harvested using cardiac puncture. Inflammatory monocytes (CD45^high^, CD115^high^, CD11c^low^ and Ly6c^high^) and patrolling monocytes (CD45^high^, CD115^high^, CD11c^low^, Ly6c^low^) were isolated from whole blood by FACS.

To study the effect of glioma on the infiltrating innate immune cells, we implanted a syngeneic glioma cell line, GL261, into C57BL6.CCR2^RFP/WT^ mouse brains (Fig. 1a). These cells express a palmitoylated form of GFP, which results in anchoring of GFP into the inner leaflet of all cellular membranes^22^ (Fig. 1b). We used the cellular uptake of membrane (particle) associated GFP as a marker for interaction between the glioma and monocytes/macrophages. The tumor was grown for 30 days after which the brain and the blood of the mice were harvested. Blood-derived monocytes were isolated based on the expression of CD45, CD115, absence of CD11c and level of Ly6C to separate Ly6C^low^ patrolling monocytes and Ly6C^high^ infiltrating monocytes (Fig. 1c)^13^. Focusing on the infiltrating myeloid cells, we isolated monocytes and macrophages using antibodies to CD11b, CD45, Ly6C and F4/80^11,23^ and fluorescence activated cell sorting (FACS), with subsequent separation based on the presence of glioma-derived GFP (Fig. 1d). Infiltrating cells were separated from microglia based on high CD11b and CD45 expression. In control brains, lacking a tumor, this CD45^high^ Cd11b^high^ population of infiltrating myeloid-derived cells was absent (Supplementary Fig. S1). Taken together, FACS using a panel of myeloid cell surface markers defined 8 different populations of blood-circulating and tumor-infiltrating cells subdivided based on the uptake of glioma-derived membrane-bound GFP.

### Global analysis of gene expression

**Figure 2:**
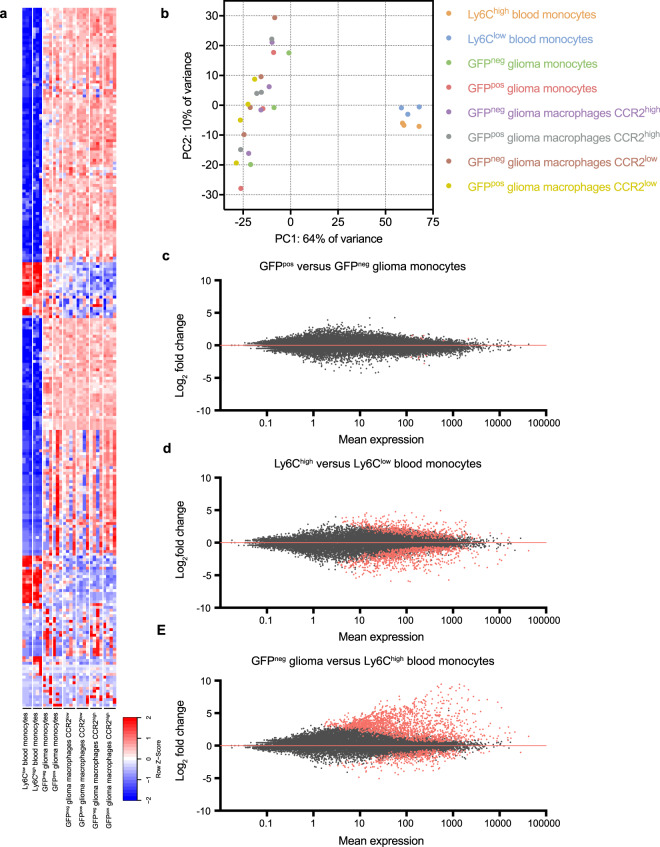
Global analyses of gene expression. (**a**) Top 250 most differentially expressed genes among 8 cell subtypes isolated from blood and brain. (**b**) PCA plot illustrates difference in principle components between brain and blood samples. (**c**) MA plot reveals the number of genes with a significant change in expression in comparison between GFP^pos^ and GFP^neg^ brain monocytes. (**d**) Significantly differentiated gene expression shown in MA plot between blood-derived classical infiltrating monocytes (Ly6C^high^) and patrolling monocytes (Ly6C^low^). (**e**) Significantly differentiated gene expression shown in MA plot between blood-derived monocytes and glioma monocytes.

To analyze the difference in gene expression between the 8 different groups, the top 250 most differentially expressed genes showed a large variation between blood and brain-derived populations (Fig. 2a). This was further confirmed by plotting the samples using a principle components analysis (PCA). Here we show large variations between blood and brain cells, while the difference within the brain samples is less prominent (Fig. 2b). To analyze the effect of tumor interaction by presence of GFP membranes within glioma monocytes, we compared the overall gene expression of GFP^pos^ and GFP^neg^ brain monocytes. Interestingly, only 11 genes were expressed at significantly different levels between the groups, indicating that the uptake of glioma-derived membrane-bound GFP does not notably change the transcriptome of these cells (Fig. 2c). Comparison of the transcriptome of the blood Ly6C^high^ and Ly6C^low^ monocytes revealed a total of 1292 significantly differentially expressed genes (Fig. 2d). Even more prominent were the changes between monocytes in the blood compared to those in the brain, as the differences between GFP^neg^ glioma monocytes and Ly6C^high^ blood-derived monocytes showed 1621 significantly upregulated genes and 398 significantly downregulated genes (Fig. 2e**)**. These results indicate that upon exiting the blood circulation, Ly6C^high^ classical infiltrating monocytes change their gene expression profile dramatically. Since the uptake of membrane-bound GFP resulted in very similar RNA expression patterns in CCR2^high^ and CCR2^low^ glioma macrophages, we decided to focus subsequent analyses on the GFP^neg^ subpopulations only.

### Expression of specific innate immune cell markers

To validate whether the populations of cells we isolated express specific innate immune cell markers, we plotted the normalized read count of these selected markers. First, to make sure the gene expression patterns reflected the corresponding protein levels, we analyzed the correlation between mean fluorescent intensity as measured by FACS and the normalized RNA read count. Plotting the LY6C protein expression in mean fluorescent intensity (MFI) compared to RNA expression of the *Ly6c2* gene, we found a Spearman’s rank correlation coefficient of 0.7177 (Supplementary Fig. S2). F4/80 is the second marker for which sufficient data was available and this marker showed a Spearman’s rank correlation coefficient of 0.7527 between protein and mRNA levels (Supplementary Fig. S2). Together this supports the concept that differences in RNA expression are, in general, representative of the corresponding protein levels.

**Figure 3:**
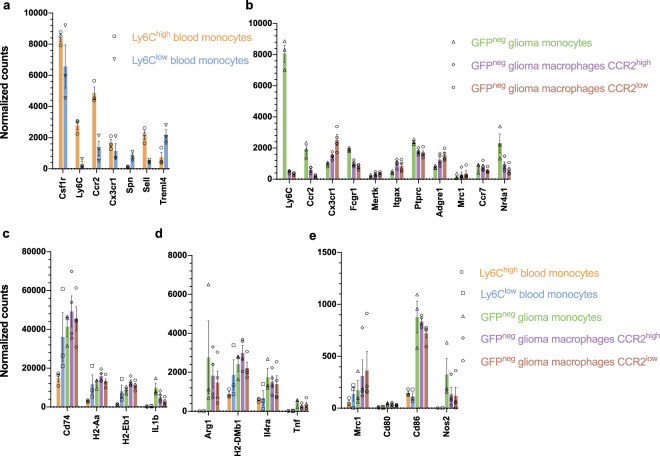
Different cell populations show expression of specific markers. (**a**) Gene expression of markers specific to inflammatory Ly6C^high^ (*Cx3cr1*^*mid*^ *Ccr2*^+^ *Cd62l*^+^ *Cd43*^*low*^ *Treml4*^+^) and patrolling Ly6c^lo*w*^ (*Cx3cr1*^*high*^ *Ccr2*^*-*^ *Cd62l*^*-*^ *Cd43*^*high*^ *Treml4*^*-*^) monocytes (**b**) Normalized read counts of cell markers used to identify and differentiate monocytes from macrophages (CCR2^high^ and CCR2^low^), including *Ly6C, Ccr2, Cx3cr1, Fcgr1, Mertk, Itgax, Ptprc, Adgre1, Mrc1, Ccr7* and *Nr4a1*. (**c**) Expression of activation markers *Cd74*, *H2-Aa*, *H2-Eb1*, *IL1b* (**d**) *Arg1*, *H2-DMb1*, *Il4ra*, *Tnf* (E) *Mrc1*, *Cd80*, *Cd86* and *Nos2* shows that after infiltration into the glioma monocytes and macrophages are in an activated state. Data represents 3 independent experiments and are presented as the mean with SEM (error bars). Differential expression analysis with fold_2_change, SEM and adjusted p-values are listed in Supplementary Table S1.

Next, we analyzed specific markers present in the blood-derived monocyte populations. Both the Ly6C^high^ and Ly6C^low^ monocytes are CSFR1^high^ and can be further characterized by the expression of the markers CX3CR1, CCR2, CD62L, CD43 and TREML4. Specifically the two populations are either LY6C^high^ CX3CR1^mid^ CCR2^high^ CD62L^high^ CD43^low^ TREML4^high^ inflammatory monocytes or LY6C^low^ CX3CR1^high^ CCR2^low^ CD62L^low^ CD43^high^ TREML4^low^ patrolling monocytes^13^. Comparing the RNA expression of these markers confirmed that the two populations analyzed represent these monocyte populations as isolated using FACS (Fig. 3a). Moreover, glioma monocytes and macrophages can be identified based on the expression of *Ly6c*, *Ccr2*, *Cx3cr1*, *Cd64*, *Mertk*, *Cd45*, *F4/80*, *Ccr7* and transcription factor *Nr4a1*^13^. Again, the expression profiles of the different cell types match the expression trends among the different populations, as described in the literature (Fig. 3b). To investigate the function of these different cell groups we plotted the expression of specific activation markers. Markers associated with an anti-inflammatory response, such as *Arg1*, *Mrc1* and *Il4ra*^24^, are expressed at a higher level in glioma-infiltrating cells, as compared to blood-derived cells (Fig. 3d–e). Similarly, MHC molecules including *H2-Aa*, *H2-Eb1* and *H2-DMb1*, showed a higher normalized read count in glioma-infiltrating cells, as compared to blood-derived cells (Fig. 3c–d). Interestingly, the gene expression of markers connected to a pro-inflammatory response, *Il1b* and *Nos2*, were also detected at high levels in glioma-infiltrating cells^25,26^ (Fig. 3c–d). Lastly, T-cell co-stimulatory molecules *Cd80* and *Cd86* were expressed at low levels in all these different cell populations (Fig. 3e)^27^. Differential expression analysis comparing all groups of all markers with log_2_fold change, SEM and adjusted p-values are listed in Supplementary Table S1. Taken together, the different cell types expressed cell-specific markers at expected levels while the glioma-infiltrating cells were found to be in an activated state compared to blood-derived cells, as shown by the higher expression of individual activation markers. These types of markers are used to predict the function of the cells, in which monocytes are known to be activated by IL10 inducing a more regulatory (or anti-inflammatory) role whereas stimuli by IFNy results in pro-inflammatory phenotype. The expression of pro- and anti-inflammatory genes in glioma-infiltrating cells illustrates the complexity of the cell polarization, and the inability to characterize these cells according to binary M1/M2 models, which have been mostly studied *in vitro*^26,28^.

### Analysis of various cytokine pathways in infiltrating monocytes indicates upregulation of both pro- and anti-inflammatory pathways

**Figure 4:**
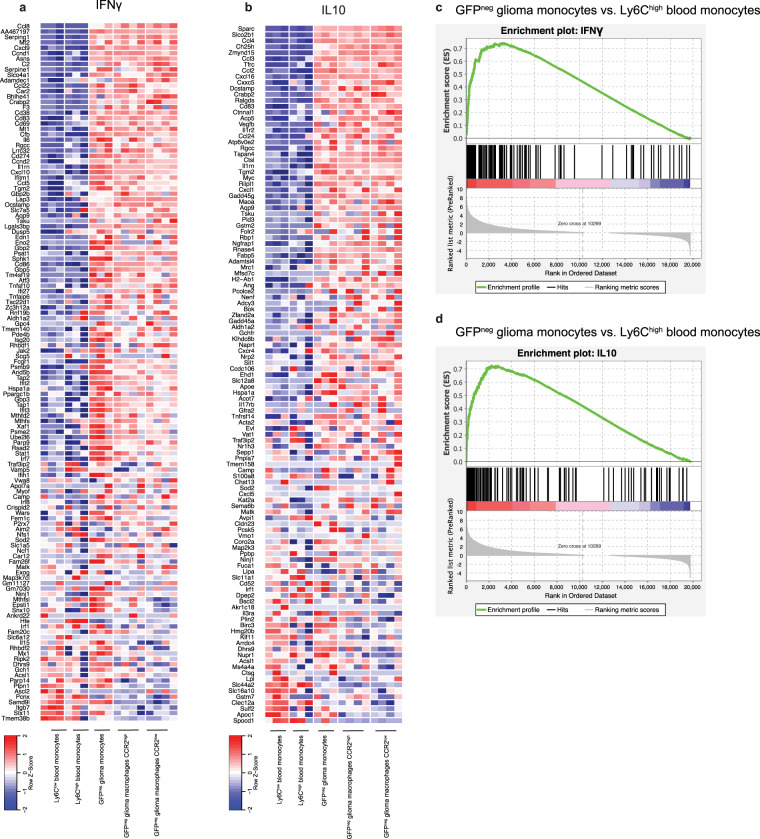
Analysis of various cytokine pathways in glioma monocytes and macrophages indicated upregulation of both pro- and anti-inflammatory pathways. (**a**) Relative expression of IFNγ related genes in the pro-inflammatory pathway, showing an overall upregulation in glioma-infiltrating cells of the IFNγ pathway. (**b**) IL10 pathway, which is anti-inflammatory, is upregulated as shown by the high relative expression in glioma-infiltrating cell groups. (**c**,**d**) Gene set enrichment analysis (GSEA) for the ranked genes based on the differential expression of genes comparing GFP^neg^ glioma monocytes to Ly6C^high^ blood monocytes, identified significant upregulation (FDR p-value < 0.05) of the IFNγ and IL10 pathways.

To further confirm that *in vivo* glioma monocytes and macrophages express a phenotype including expression of both pro- and anti-inflammatory genes, we looked at cytokine-associated gene sets. Here, we focused on the expression of IFNγ, IL10, IL6/STAT3 and IL4 associated gene sets. In line with the pattern of individual markers, we found upregulation of both pro- and anti-inflammatory pathways. IFNγ associated genes, a pro-inflammatory pathway, were upregulated in brain monocytes and macrophages compared to monocytes in the blood (Fig. 4a). Additionally, IL4 and IL10 (anti-inflammatory) and IL6/STAT3 (pro- and anti-inflammatory) associated genes were also significantly upregulated in monocytes and macrophages in a glioma-bearing brain (Fig. 4b–d & Supplementary Fig. S3)^29^. This confirms published data that *in vivo* monocytes and macrophages have a much more complex phenotype than the initially proposed binary M1/M2 model^28^.

### Web-based tool for analyzing circulating and glioma-infiltrating monocytes and macrophages in glioma model

**Figure 5:**
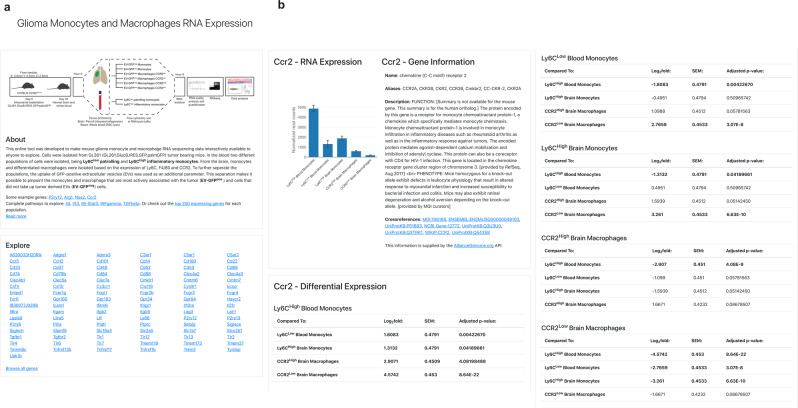
Web-based tool for studying circulating and glioma monocytes and macrophages in glioma model. (**a**) On the homepage of website (www.glioma-monocytes.com) the experimental setup is summarized as a schematic and in text. Expression of individual genes in various cell populations can be consulted using the search bar. (**b**) Example of output of inquiry focusing on Ccr2, showing normalized read count and differential expression between all cell groups.

To combine the extensive datasets gathered in sequencing the transcriptome of the above-described monocyte and macrophage populations we set up a web-based tool at www.glioma-monocytes.com. This user-friendly platform allows users to browse individual genes (Fig. 5a). Here, the expression level of all individual genes within the different populations are displayed, supplemented with differential expression data among the populations (Fig. 5b). Additional information has been extracted from the AllianceGenome API to display functional data about the gene of interest as well as links to external databases. This website will help researchers investigate properties of circulating monocytes, glioma-infiltrating monocytes and differentiated macrophages in an orthotopic mouse glioma model.

## Discussion

In this study we have analyzed the transcriptome of different monocyte subtypes derived from blood and tumor tissue. We found that by using a set of specific cell surface markers, we were able to separate blood monocytes, tumor-localized monocytes and macrophages in a glioma mouse model. Here, we have developed a user-friendly, freely available online tool to study the gene expression of different tumor-infiltrating and blood-derived monocyte cell populations.

Overall, a high level of differential gene expression was observed between these different cell subtypes, most notably between blood-derived and glioma-infiltrating cells. Although the global gene expression of the two different monocyte subtypes isolated from blood was similar as compared to all the other subtypes, the difference between these monocyte subpopulations was distinguished by analyzing specific cell markers, such as *Cx3cr1*, *Cd64*, *Cd43* and *Treml4*, as previously reported^13^. Similarly, the subtypes isolated from tumor tissue showed expression patterns of *Ly6C*, *Ccr2*, *Fcgr1*, *F4/80* and *Nr4a1* which was characteristic of infiltrating monocytes and macrophages shown in previous reports^13^. Both the glioma-infiltrating monocytes and macrophages had pro- (*Arg1*) and anti-inflammatory (*Nos1*) markers, which is in line with the global upregulation expression of IFNγ, IL10, IL4 and IL6/STAT3 cytokine pathway genes, albeit it is not clear if individual cells express both pro- and anti-inflammatory marker. Thus, glioma monocytes and macrophages *in vivo* cannot be characterized as either M1 or M2, but collectively express a more complex phenotype that shares characteristics of both these two differentiation states. This was also shown using single cell RNAseq of CD11b^+^ TAMs from human glioma biopsy samples, where both M1 and M2 markers were found to be co-expressed on individual TAMs^30^.

We have sought to use the GFP-membrane markers expressed by tumor cells to identify glioma monocytes and macrophages with a high level of interaction with the glioma. However, differential expression analysis did not show substantial variation between the subtypes of monocytes and macrophages, which were positive or negative for GFP. A possible explanation is that tumors secrete a plethora of cytokines and chemokines. These factors may be the dominant determinants in the activation and differentiation of infiltrating monocytes and macrophages regardless of whether they took up tumor-derived material or not.

Increasing evidence indicates that TAMs play a crucial role in brain tumor development. Understanding how monocytes differentiate into macrophages and identification of new specific markers is needed to give insight into how these cells support or inhibit cancer progression. So far, targeting of TAMs has only had limited success. Two properties of TAMs have been exploited in therapy: the recruitment and reprogramming. Colony stimulating factor 1 receptor (CSF1R) has been shown to target both venues^31,32^. For example, depletion of TAMs by blocking CSF1R, has been shown to increase survival by 64.3% in a mouse pro-neural GB model^31^. However, subsequent studies found development of resistance to the CSF1R blockage through IGF1 secretion by TAMs resulting in resumed PI3K dependent tumor growth^32^. While a number of studies targeting CSF1R are still in progress (Clinical Trial Identifier: NCT02829723 and NCT01790503), in a phase II clinical trial using an inhibitor for CSF1R, PLX3397, it was shown that the drug was tolerated and can cross the blood-brain barrier, but did not show any efficacy^33^. The lack of efficacy underlines the need for a better understanding of TAMs.

Here we have used the mouse glioma cell line GL261 in a syngeneic model to investigate the changes in the transcriptome of infiltration monocytes as well as circulating monocytes. While this model is widely used, is has the limitations that most mouse models have. First, the growth pattern of GL261 is not diffuse infiltrative, as observed in human gliomas. Secondly, while GL261 harbors a TP53 mutation often detected in glioblastomas, it lacks the classical IDH wildtype glioblastoma molecular associated alterations such as PTEN, PI3K and TERT promotor mutations, EGFR alterations as well as chromosomal alterations such as gain of chromosome 7, loss of chromosome 10 and homozygous deletion of CDKN2A/CDKN2B^34,35^. To counter these issues, one could add additional murine glioma cell lines such as CT2A, however also these alternative lines do not recapitulate human GB in multiple aspects. Moreover, the use of a xenograft model would completely alter the immune response to the tumor, a component of which (monocytes and macrophages) we are focusing on. Therefore, this study provides an exploratory tool based on the data acquired from one murine cell line, with the limitations outlined above, that can help to find targets of interest that require additional validation in other cell lines or preferable human glioblastoma data.

Glioma is a complex disease and the composition of the tumor environment, including glioma-infiltrating monocytes and macrophages, as well as microglia (Maas, Abels *et al*.) has a substantial effect on tumor growth and response to therapy^36^. To elucidate the role of these cells in glioma we have aimed to decipher the molecular profile of these stromal cells. While this can give insight into potential targets and markers for these monocyte subtypes, our incomplete understanding still hinders the development of stromal-targeted therapeutics and thus future research will be needed to address the relationship between and the function of these different cell types.

## Methods

### Cell culture

GL261 cells (NCI Tumor Repository) were cultured in Dulbecco’s modified Eagle’s medium (DMEM) (Corning) with penicillin (100 units/ml) and streptomycin (100 μg/ml) (P/S) (Corning) and 10% fetal bovine serum (FBS) (Gemini Bioproducts). Cells were cultured in a 5% CO_2_ humidified incubator at 37 °C. Cells were periodically tested for mycoplasma contamination and found negative. Reporter genes (palmGFP and GlucB-GFP) were introduced in GL261 by lentiviral transduction creating GL261.BpalmGFP cells^22,37^.

### Mice

Animal experiments were conducted under the oversight of the Massachusetts General Hospital Institution Animal Care and Use Committee. Animal protocols were approved by the Institutional Animal Care and Use Committee (IACUC) for the Massachusetts General Hospital (MGH) following the guidelines of the National Institutes of Health for the Care and Use of Laboratory Animals. To generate heterozygous C57BL6.CCR2^RFP/WT^ knock-in mice, C57BL/6 mice (Charles River Laboratories) were crossed with homozygous C57/BL6.CCR2^RFP/RFP^ knock-in mice^38^. Adult mice ranging from 12–18 weeks were used in this study. Mice were maintained under a 12-hour light/dark cycle with free access to water and food. Total of four animals, two male and two female mice were randomly assigned to experimental groups. After quality control of the sequence data we excluded samples not meeting the required quality (sample cut-off <6000 genes with <5 read/gene). This resulted in n = 3 per isolated cell type.

### Immunofluorescence

Cells were plated on coverslip pre-coated with poly-D-lysine (PDL) (100 µg/ml, Thermo Fisher). Fixation of cells was done using 4% paraformaldehyde (PFA) for 20 min at room temperature (R/T). Cells were washed using PBS following DAPI (1 μg/ml, Thermo Fisher) staining performed for 30 min at R/T. Coverslips were washed for 10 min using PBS and mounted on microscope slides using ProLong Diamond Antifade Mountant (Thermo Fisher). Zeiss Axio Imager M2 (Carl Zeiss, Oberkochen, Germany) was used to acquire fluorescence microscopy images.

### Intracranial tumor implantation

Mice were anesthetized using 70 μl ketamine (Bioniche Pharma) (17.5 mg/ml) and xylazine (Santa Cruz Biotechnology) (2.5 mg/ml). GL261.BpalmGFP (1 ×10^5^ cells in 2 µl DMEM) were implanted in the striatum using a stereotactic frame. Implantation was done at the coordinates from lambda: 2 mm anterior, 0.5 mm left and a depth of 2.5 mm. Four weeks after implantation, the mice were euthanized using a 120 μl ketamine (17.5 mg/ml) and xylazine (2.5 mg/ml). This was followed by cardiac puncture to collect blood using a syringe containing 100 μl of 5 M EDTA. After blood collection, 50 ml PBS was used for transcardial perfusion with a perfusion pump (Minipump Variable Flow, Fisher Scientific) after which the brains were collected for further processing.

### Harvesting of brains and blood and preparation of single-cell suspensions

Collected brains were first manually separated into smaller fragments and transferred to GentleMacs C-tube (Miltenyi Biotech, San Diego, CA, USA) containing Roswell Park Memorial Institute (RPMI) 1640 with L-glutamine (no phenol red) medium (Fisher Scientific) supplemented with Dispase (2 U/ml) (Corning) and Collagenase Type 3 at a final concentration of 200 U/ml (Worthington Biochemicals). The brains were mechanically dissociated using the GentleMACS Dissociator (Miltenyi Biotech) pre-set brain program 1 and 2 with intervals of 10 min incubation at 37 °C for 10 min. Finally, DNase I grade II (Roche Applied Science) was added to a concentration of 40 U/ml incubated at 37 °C for 10 min following brain program 3. Brain suspension was filtered and transferred using 100 µm cell strainer into 50 ml Falcon tube and centrifuged at 400 x g for 10 min. Cell pellets were resuspended in 10.5 ml RPMI/L-glutamine, mixed gently with 4.5 ml physiologic Percoll (Sigma Aldrich) and centrifuged at 850 × g without brake for 40 min. Pellet was washed using PBS and centrifuged at 400 x g for 10 min. Red blood cells in final pellet were lysed using RBC lysis (Boston BioProducts) for 2 min at R/T. Cells were washed twice with PBS.

Blood was subjected to red blood cell lysis using RBC lysis (Boston BioProducts) for 2 min at R/T. Cells were washed twice with PBS without Mg^2+^ and Ca^2+^ (Corning). Remaining cells were washed twice with DMEM (Corning) supplemented with 5 mM EDTA and 0.5% BSA and finally pelleted by centrifugation at 250 × g for 10 min.

The final brain and blood cell suspensions were resuspended in 300 µl PBS with 0.2% FBS, followed by staining for FACS.

### Cell staining and FACS

Prior to antibody staining, cells were incubated for 10 min on ice with TruStain fcX (anti-mouse CD16/32, BioLegend #101319, clone 93, 1:100). To identify infiltrating monocytes/macrophages from the brain, anti-CD11b-PE-Cy7 (BioLegend, M1/70, 1:100), anti-CD45-pacificBlue (BioLegend, 30-F11, 1:100), anti-F4/80-APC (BioLegend, BM8, 1:75) and anti-Ly6C-BV605 (BioLegend, HK1.4, 1:500) were used. Blood-derived monocytes were identified using anti-CD45-pacificBlue (BioLegend, 30-F11), anti-Ly6C-BV605 (BioLegend, HK1.4, 1:100), anti-Cd115-APC (BioLegend AFS98, 1:100) and anti-Cd11c-APC-Cy7 (BioLegend, N418, 1:100). Cells were stained by incubation with antibodies for 30 min on ice. Finally, cells were washed using 1 ml PBS and centrifuged at 400 × g for 8 min. Cell pellets were resuspended in 300 µl PBS supplemented with 0.2% FBS and filtered through a 35 µm cell strainer (BD Falcon). Cell subpopulations were finally sorted into RLT buffer (Qiagen) using a BD FACSAria II SORP Cell Sorter.

### RNA isolation and preparation for RNA-sequencing

Cells were sorted into 350 μl RLT Plus lysis buffer (Qiagen) at 4 °C for direct cell lysis. RNA isolation was carried out using the RNeasy Plus Micro kit (Qiagen) following the total RNA isolation protocol (appendix D). RNA concentrations and quality (RIN) were analyzed on pico-chips using the Agilent 2100 Bioanalyzer (Agilent Technologies). Library preparation was done with SMARTer cDNA protocol in addition to Nextera XT DNA Library Preparation kit. First, reverse transcription of 500 pg RNA into cDNA was done with the SMARTer Ultra Low Input RNA Kit for Sequencing – v3 (Clontech Takara) using 3’-SMART CDS primer II A (selecting for poly-A transcripts), according to the manufacturer’s protocol. ERCC RNA Spike-In Mix (Life Technologies) was added prior to reverse transcription. cDNA was purified with 1x Agencourt AMPure XP beads (Beckman Coulter), following the SMARTer protocol. Subsequently barcoding and fragmentation of cDNA was done using Nextera XT DNA Library Preparation kit (Illumina). cDNA (1 ng) was used as input for the enzymatic tagmentation and PCR amplification (12 cycles). Final PCR product was purified with 1.8x Agencourt AMPure XP beads as described in the Nextera XT protocol, without the bead-based library normalization step. Library validation and quantification was done using the SYBR FAST Universal qPCR Kit (KAPA Biosystems). The individual libraries were pooled in equal molar concentrations, and the pool concentration was determined again using the KAPA SYBR FAST Universal qPCR Kit. The library pool was subsequently diluted, denatured, and loaded onto the NextSeq. 500 sequencer (Illumina) with the addition of 1% PhiX Sequencing Control V3 (Illumina). Sequencing was done using NextSeq. 500/550 High Output v2 kit (150 cycles) with 75-bp paired-end sequencing.

### Data processing and statistical analysis

Raw data was aligned, duplicates removed and counted. First alignment was done against mm10 genome using the STAR v2.4.0 h aligner set at default. All duplicate reads were marked and removed using the MarkDuplicates tool in picard-tools-1.8.4. Finally, aligned reads were counted against Gencode’s GRCm38.p3 GTF annotations using htseq-count in the intersection-strict mode. Readcount files were generated with HTSeq-count version 0.6.1p1.

Data analysis of mapped counts was performed in R 3.2.3 using the DESeq. 2 package (version 1.10)^39^. For unsupervised clustering, sample read counts were normalized using the regularized logarithm transformation method^39^. The regularized logarithm (rlog) values were used to plot heatmaps using the gplots (version 2.17) heatmap.2 function in R. Unsupervised clustering was performed based on the top-250 most variable genes between samples. Differential expression analysis was performed in DESeq. 2 and only two-sided Benjamini and Hochberg multiple testing adjusted p-values are reported in this manuscript. The level of significance used is <0.05 Benjamini and Hochberg multiple testing adjusted p-value. Error bars display mean ±standard error of the mean (SEM). The “n” represents three individual mice.

For analysis of specific gene sets, the microglial sensome was extracted from Hickman *et al*. 2013^40^. The IL6/STAT3 and TGF-β sets were extracted from the Gene set enrichment analysis (GSEA) hallmarks collection^41^. The IL4, IL10 and IFNγ sets were calculated from the Xue *et al*.^42^. study by extracting the 150 highest upregulated genes compared to baseline. For the IL6/STAT3, TGF-β, IL4, IL10 and IFNγ sets, human to mouse homolog conversions were performed using The Jackson Laboratory Human and Mouse Homology Report (accessed February 18^th^, 2016) supplemented by manual curation. Principal component analysis (PCA) was performed by utilization of the DESeq. 2’s built-in PCA function using the default settings. Final bar graph, dotplots, PCA and MA plots were generated in GraphPad Prism (version 7.02). GSEA was performed using the graphical user interface for Mac OSX version 4.0.3. build 23 using the GSEA pre-ranked module using the default settings.

## Supplementary information

Supplementary Information.

## Data Availability

Raw and processed data were deposited in NCBI’s Gene Expression Omnibus (GEO) and are accessible using GSE145506 at https://www.ncbi.nlm.nih.gov/geo/query/acc.cgi?acc=GSE145506. Reviewer token: qzulwcikbrsppgv.

## Code availability

All R scripts written for data processing and the generation of figures included in this manuscript are available online in a git repository. The files and information can be accessed at: https://github.com/slnmaas/MM2-Project.
