# Supplementary material for: GlioM&M: Web-based tool for studying circulating and infiltrating monocytes and macrophages in glioma
Source: Sci Rep. 2020 Jun 18;10:9898. doi: 10.1038/s41598-020-66728-w (PMC7303151; doi:10.1038/s41598-020-66728-w)
Supplement: Supplementary file 1 — Supplementary Information. [file 41598_2020_66728_MOESM1_ESM.pdf]

GlioM&M: Web-based tool for studying circulating and infiltrating monocytes and macrophages in glioma

Erik R Abels[1Ω], Sybren L.N. Maas[2Ω], Eric Tai[3], David T. Ting[3], Marike L.D. Broekman[4,5], Xandra O. Breakefield[1], and Joseph El Khoury[6ϕ*]

[1] Departments of Neurology and Radiology, Massachusetts General Hospital, and Harvard Medical School, Boston, 02129, Massachusetts, USA

[2] Department of Neurosurgery, UMC Utrecht Brain Center, University Medical Center, Utrecht University, 3584 CX, Utrecht, The Netherlands

[3] Cancer Center, Massachusetts General Hospital, and Harvard Medical School. Boston, 02114, Massachusetts, USA

[4] Department of Neurosurgery, Leiden University Medical Center, 2300 RC, Leiden, The Netherlands

[5] Department of Neurosurgery, Haaglanden Medical Center, 2512 VA, The Hague, The Netherlands

[6] Center for Immunology & Inflammatory Diseases, Massachusetts General Hospital, and Harvard Medical School. Boston, 02129, Massachusetts, USA.

[Ω] Shared co-authors

[ϕ] Lead contact

[*]Correspondence: jelkhoury@mgh.harvard.edu

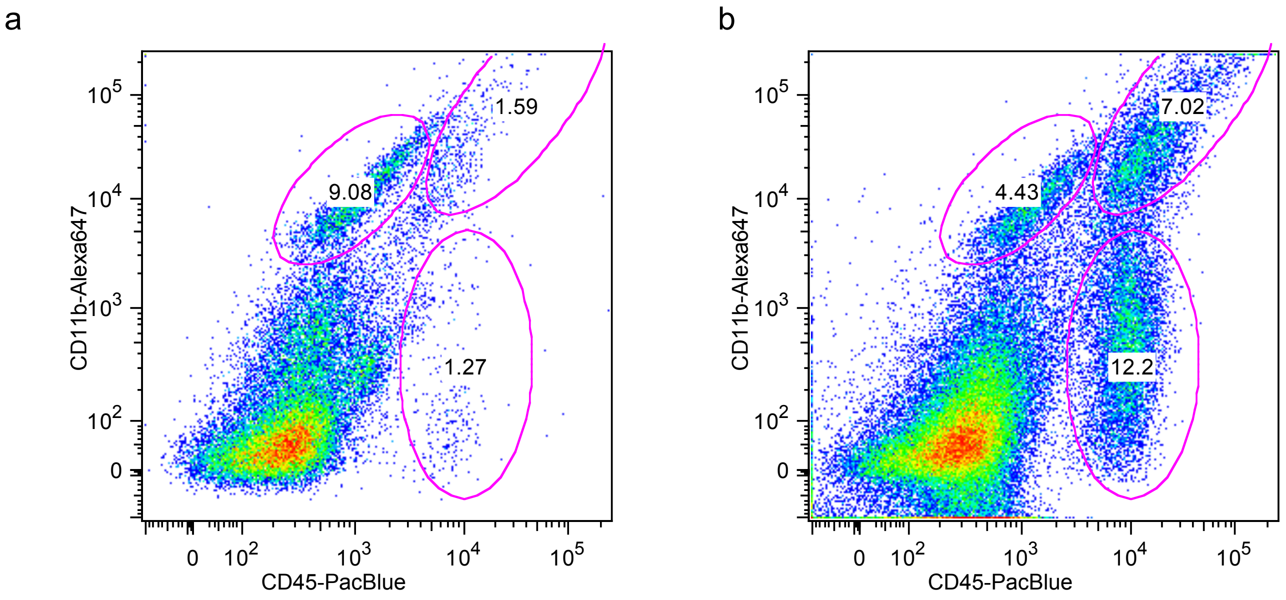

Supplementary Figure S1. Monocytes infiltrate brain upon tumor implantation. (**A**) Expression of CD11b and CD45 in control brain shows one dominant population of cells with intermediate expression of CD45 characterized as microglia. (**B**) Presence of tumor in brain results in the influx of CD11b^{high}, CD45^{high} glioma monocytes/macrophages.

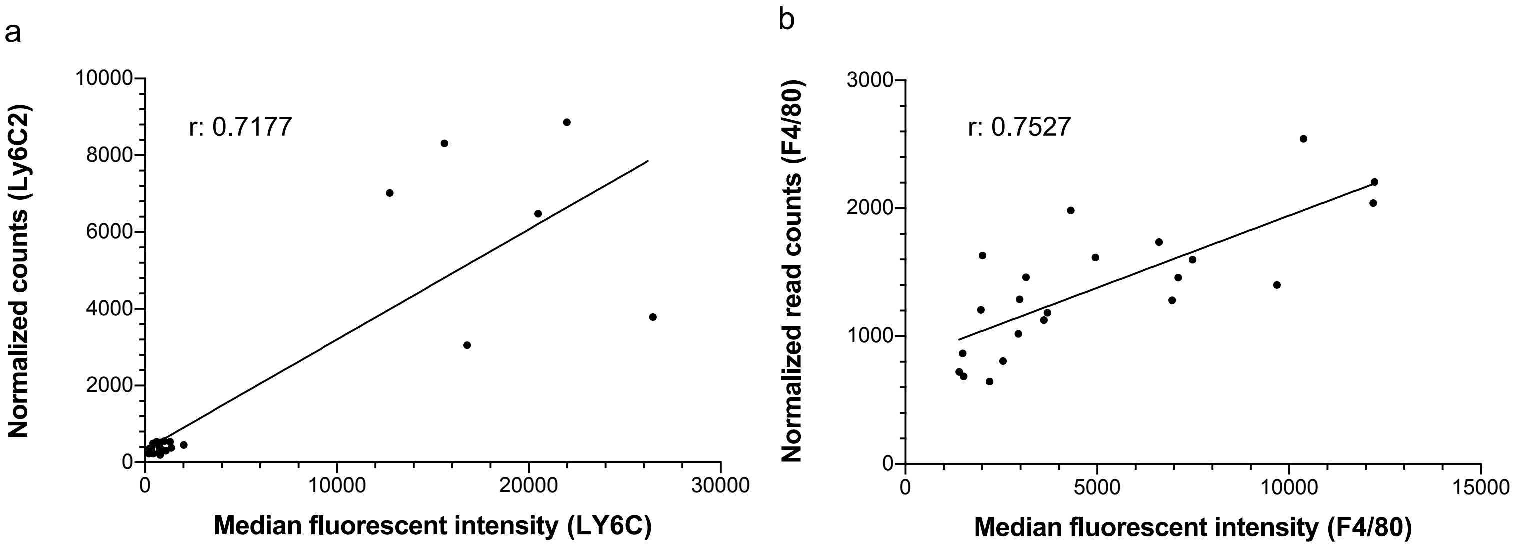

Supplementary Figure S2. RNA expression is correlated with protein levels in isolated cells. (**A**) Normalized counts and median fluorescent intensity of Ly6C in all cell populations shows high level of nonparametric Spearman correlation (r: 0.7177). (**B**) Normalized counts and median fluorescent intensity of F4/80 in all cell populations is consistent with high level of nonparametric Spearman correlation (r: 0.7527).

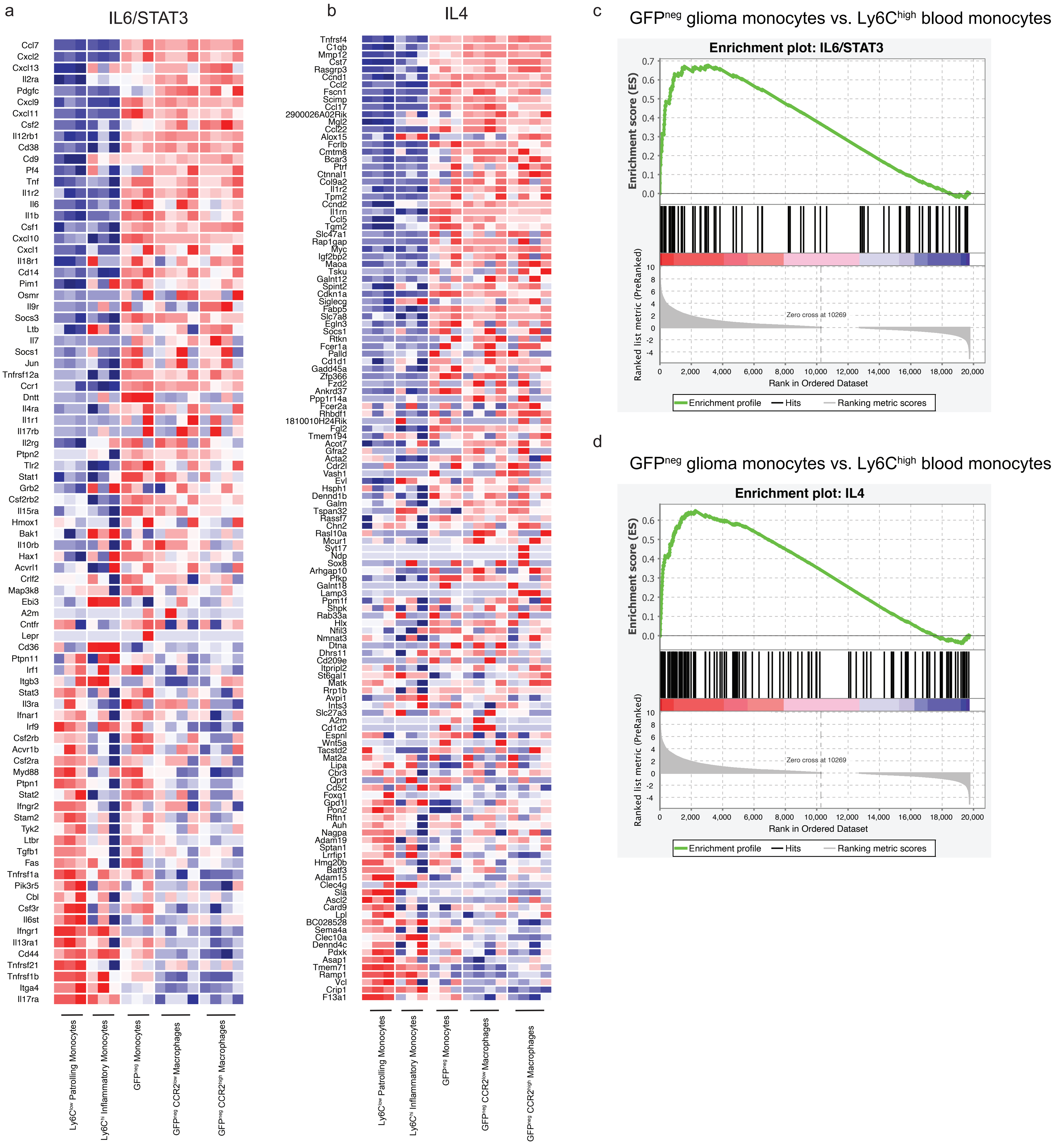

Supplementary Figure S3. Analysis of IL6/STAT3 and IL4 cytokine pathways in glioma monocytes and macrophages versus blood-derived monocytes shows pro-and anti-inflammatory pathway activation. (**A**) IL6/STAT3 pathway, a pathway associated with pro- and anti-inflammatory properties, is upregulated as shown by the high relative expression in tumor infiltrating cell groups. (**B**) Genes associated with the anti-inflammatory IL4 pathway are expressed at higher level in monocytes and macrophages found in tumor compared to blood-derived monocytes. (**C and D**) Gene set enrichment analysis (GSEA) for the ranked genes based on the differential expression of genes comparing GFPneg glioma monocytes to Ly6Chigh blood monocytes, identified significant upregulation (FDR p-value <0.05) of the IL6/STAT3 and IL4 pathways.

Supplementary Table S1. Differential expression analysis of the specific markers in the different cell populations.

	Ly6C^{High} Blood Monocytes compared to Ly6C^{Low} Blood Monocytes		
	Log$_2$fold:	SEM:	Adjusted p-value:
Csf1r	0.3505	0.2679	0.447625
Ly6C	3.1203	0.4835	3.135212
Ccr2	1.8083	0.4791	0.004227
Cx3cr1	0.5116	0.3771	0.426897
Spn	-2.656	0.4288	1.303453
Sell	2.1469	0.4704	0.000277
Treml4	-1.4378	0.667	0.15192

Supplementary Table S1A. Differential expression analysis of Ly6C^{High} Blood monocytes compared to Ly6c^{Low} blood monocytes with fold$_2$change, SEM and adjusted p-values, corresponding to Figure 3A. (Significant genes in grey: p-adjusted p-value <0.05)

	Ly6C^{High} Brain Monocytes compared to CCR2^{High} Brain Macrophages			Ly6C^{High} Brain Monocytes compared to CCR2^{Low} Brain Macrophages			CCR2^{High} Brain Macrophages compared to CCR2^{Low} Brain Macrophages		
	Log$_2$fold:	SEM:	Adjusted p-value:	Log$_2$fold:	SEM:	Adjusted p-value:	Log$_2$fold:	SEM:	Adjusted p-value:
**Ly6C**	3.9043	0.4539	9.10E-14	4.5142	0.4546	3.25E-19	0.6099	0.4248	1
**Ccr2**	1.5939	0.4512	0.051425	3.261	0.4533	6.63E-10	1.6671	0.4232	0.086785
**Cx3cr1**	0.5967	0.3544	0.891859	-1.3438	0.3542	0.010555	-0.7471	0.3286	1
**Fcgr1**	0.9251	0.2164	0.00559	1.2782	0.217	1.49E-06	0.3531	0.2016	1
**Mertk**	-0.735	0.4429	0.906502	-0.9905	0.4428	0.314565	-0.2555	0.4098	1
**Itgax**	-1.1709	0.5106	0.529809	-0.9344	0.5108	0.525399	0.2364	0.4758	1
**Ptprc**	0.438	0.226	0.753159	0.5202	0.2261	0.285221	0.0822	0.2097	1
**Adgre1**	-0.6781	0.2639	0.393226	-0.9771	0.2639	0.013806	-0.2991	0.2437	1
**Mrc1**	-0.7276	0.776	1	-0.9197	0.7761	0.830959	-0.1921	0.7326	1
**Ccr7**	-0.1238	0.5257	1	0.3798	0.5261	0.966009	0.5037	0.4911	1
**Nr4a1**	1.2849	0.5065	0.410608	1.8326	0.5068	0.01747	0.5477	0.4733	1

Supplementary Table S1B. Differential expression analysis of Ly6C^{High} Brain Monocytes compared to CCR2^{High} Brain Macrophages, Ly6C^{High} Brain Monocytes compared to CCR2^{Low} Brain Macrophages and CCR2^{High} Brain Macrophages compared to CCR2^{Low} Brain Macrophages with fold$_2$change, SEM and adjusted p-values, corresponding to Figure 3B. (Significant genes in grey: p-adjusted p-value <0.05)

	Ly6C^{High} Blood Monocytes compared to Ly6C^{Low} Blood Monocytes			Ly6C^{High} Blood Monocytes compared Ly6C^{High} Brain Monocytes			Ly6C^{High} Blood Monocytes compared CCR2^{High} Brain Macrophages			Ly6C^{High} Blood Monocytes compared CCR2^{Low} Brain Macrophages		
	Log_2fold:	SEM:	Adjusted p-value:	Log_2fold:	SEM:	Adjusted p-value:	Log_2fold:	SEM:	Adjusted p-value:	Log_2fold:	SEM:	Adjusted p-value:
**Cd74**	-1.2353	0.3948	0.02385	-1.4252	0.3948	0.00361	-1.6726	0.3706	9.41E-5	-1.5568	0.3706	0.0003
**H2-Aa**	-1.8863	0.3794	5.21E-5	-1.9483	0.3794	7.50E-06	-2.3204	0.3561	2.68E-9	-2.0389	0.3561	2.38E-07
**H2-Eb1**	-2.583	0.365	7.72E-10	-2.7149	0.3651	8.45E-12	-3.2453	0.3425	3.62E-19	-3.0179	0.3425	1.17E-16
**IL1b**	-0.7593	0.5374	0.40318	-5.2504	0.5364	2.86E-20	-4.3642	0.5053	5.41E-16	-3.5561	0.5054	8.57E-11
**Arg1**	-0.5536	0.8529	0.76673	-9.1603	0.8197	1.78E-26	-8.674	0.7849	5.36E-26	-8.3941	0.785	2.22E-24
**H2-DMb1**	-1.0238	0.435	0.11017	-1.3805	0.4351	0.01357	-1.6863	0.4086	0.00045	-1.2646	0.4087	0.01235
**Il4ra**	-0.2289	0.5655	0.88451	-1.4953	0.5653	0.05265	-1.3113	0.5327	0.06464	-1.199	0.5327	0.09464
**Tnf**	-0.6023	0.719	0.67171	-4.3461	0.711	4.07E-08	-3.5857	0.675	2.35144	-3.969	0.6749	1.02E-07
**Mrc1**	-1.1392	0.8178	0.41148	-1.4336	0.8181	0.27374	-2.1612	0.778	0.03073	-2.3533	0.778	0.01507
**Cd80**	0.1315	0.6455	0.97052	-2.1759	0.6153	0.0046	-1.8439	0.5827	0.01106	-1.7248	0.5856	0.01859
**Cd86**	0.367	0.3352	0.54922	-2.5304	0.3293	1.48E-12	-2.4644	0.3091	1.15477	-2.2531	0.3095	1.63E-11
**Nos2**	0	0.9855	1	-6.5559	0.9409	2.03E-10	-5.5169	0.9057	3.42978	-5.373	0.9061	7.68E-08

Supplementary Table S1C. Differential expression analysis of Ly6C^{High} Blood Monocytes compared to Ly6C^{Low} Blood Monocytes, Ly6C^{High} Blood Monocytes compared Ly6C^{High} Brain Monocytes, Ly6C^{High} Blood Monocytes compared CCR2^{High} Brain Macrophages and Ly6C^{High} Blood Monocytes compared CCR2^{Low} Brain Macrophages with $fold_2$change, SEM and adjusted p-values, corresponding to Figure 3C-E. (Significant genes in grey: p-adjusted p-value <0.05)

	Ly6C^{Low} Blood Monocytes compared Ly6C^{High} Brain Monocytes			Ly6C^{Low} Blood Monocytes compared CCR2^{High} Brain Macrophages			Ly6C^{Low} Blood Monocytes compared CCR2^{Low} Brain Macrophages		
	**Log$_2$fold:**	**SEM:**	**Adjusted p-value:**	**Log$_2$fold:**	**SEM:**	**Adjusted p-value:**	**Log$_2$fold:**	**SEM:**	**Adjusted p-value:**
**Cd74**	-0.1899	0.3948	0.78662	-0.4373	0.3706	0.4217	-0.3215	0.3706	0.5721
**H2-Aa**	-0.0619	0.3792	0.93683	-0.434	0.3558	0.40257	-0.1526	0.3558	0.79754
**H2-Eb1**	-0.1318	0.3645	0.84565	-0.6623	0.3419	0.14979	-0.4348	0.3419	0.37828
**IL1b**	-4.4911	0.5353	5.44E-15	-3.6049	0.5041	4.77E-11	-2.7968	0.5042	6.63E-07
**Arg1**	-8.6068	0.7987	1.28E-24	-8.1204	0.7625	3.93E-24	-7.8406	0.7626	1.50E-22
**H2-DMb1**	-0.3566	0.4346	0.61575	-0.6624	0.4081	0.24164	-0.2408	0.4082	0.7175
**Il4ra**	-1.2664	0.565	0.09481	-1.0825	0.5324	0.12697	-0.9701	0.5325	0.1772
**Tnf**	-3.7438	0.7041	2.75E-06	-2.9834	0.6676	0.00011	-3.3667	0.6675	8.16E-06
**Mrc1**	-0.2945	0.816	0.84579	-1.022	0.7757	0.36097	-1.2141	0.7757	0.26035
**Cd80**	-2.3074	0.6137	0.00181	-1.9754	0.5809	0.00479	-1.8563	0.5838	0.00889
**Cd86**	-2.8975	0.33	2.06E-16	-2.8314	0.3098	8.06E-18	-2.6201	0.3102	2.58E-15
**Nos2**	-6.6463	0.9381	8.70E-11	-5.6073	0.9027	1.80E-08	-5.4634	0.9031	4.13E-08

Supplemental Table S1D. Differential expression analysis of Ly6C^{Low} Blood Monocytes compared Ly6C^{High} Brain Monocytes, Ly6C^{Low} Blood Monocytes compared CCR2^{High} Brain Macrophages and Ly6C^{Low} Blood Monocytes compared CCR2^{Low} Brain Macrophages with fold$_2$change, SEM and adjusted p-values, corresponding to Figure 3C-E. (Significant genes in grey: p-adjusted p-value <0.05)

	Ly6C^{High} Brain Monocytes compared CCR2^{High} Brain Macrophages			Ly6C^{High} Brain Monocytes compared CCR2^{Low} Brain Macrophages			CCR2^{High} Brain Macrophages compared CCR2^{Low} Brain Macrophages		
	**Log$_2$fold:**	**SEM:**	**Adjusted p-value:**	**Log$_2$fold:**	**SEM:**	**Adjusted p-value:**	**Log$_2$fold:**	**SEM:**	**Adjusted p-value:**
**Cd74**	-0.2474	0.3706	1	-0.1316	0.3706	0.98971	0.1158	0.3445	1
**H2-Aa**	-0.3721	0.3559	1	-0.0907	0.3559	0.99534	0.2814	0.3307	1
**H2-Eb1**	-0.5304	0.3419	0.95942	-0.303	0.3419	0.92616	0.2274	0.3176	1
**IL1b**	0.8863	0.503	0.84499	1.6943	0.5031	0.03421	0.808	0.4696	1
**Arg1**	0.4863	0.7233	1	0.7662	0.7233	0.88186	0.2799	0.6816	1
**H2-DMb1**	-0.3058	0.4082	1	0.1159	0.4083	0.99158	0.4217	0.3798	1
**Il4ra**	0.1839	0.5322	1	0.2963	0.5322	0.97838	0.1124	0.4972	1
**Tnf**	0.7604	0.6588	1	0.3771	0.6587	0.97838	-0.3833	0.6188	1
**Mrc1**	-0.7276	0.776	1	-0.9197	0.7761	0.83096	-0.1921	0.7326	1
**Cd80**	0.332	0.5464	1	0.4511	0.5496	0.94566	0.1191	0.512	1
**Cd86**	0.066	0.3035	1	0.2773	0.3038	0.9185	0.2113	0.2817	1
**Nos2**	1.039	0.85	1	1.1829	0.8503	0.7443	0.1439	0.8084	1

Supplemental Table S1E. Differential expression analysis of Ly6C^{High} Brain Monocytes compared CCR2^{High} Brain Macrophages, Ly6C^{High} Brain Monocytes compared CCR2^{Low} Brain Macrophages and CCR2^{High} Brain Macrophages compared CCR2^{Low} Brain Macrophages with fold$_2$change, SEM and adjusted p-values, corresponding to Figure 3C-E.
